# Oral Contraceptive Use and Alveolar Osteitis Following Third Molar Extraction: A Systematic Review and Meta-Analysis

**DOI:** 10.1155/2022/7357845

**Published:** 2022-11-01

**Authors:** Madison Tang, Daniela Gurpegui Abud, Jaffer A. Shariff

**Affiliations:** ^1^Columbia University College of Dental Medicine, New York, NY 10032, USA; ^2^Touro University College of Dental Medicine at New York Medical College, Hawthorne, NY 10532, USA

## Abstract

**Purpose:**

Alveolar osteitis (AO) is a common postoperative complication of third molar extractions that is thought to be associated with the intake of oral contraceptives (OCPs). This meta-analysis sought to evaluate the risk of AO associated with OCP use and sex independently and whether this risk was affected by the use of postoperative analgesics or antibiotics.

**Methods:**

PubMed/Medline, EMBASE, and Cochrane databases were searched for articles pertaining to OCP use and the incidence of AO using MESH terms. The measured outcome was the development of AO following a third molar extraction. Additional variables such as sex, analgesic, and antibiotic use were documented and included in the analysis. The data were analyzed in R using the Mantel-Haenszel method.

**Results:**

Fifteen studies with a total of 1366 female participants who were OCP users and 2919 nonuser female participants were included in this meta-analysis. OCP users were approximately twice (pooled-RR: 1.98, 95% CI: 1.42–2.76) as likely to develop AO following a third molar extraction when compared to nonuser females. The increased incidence of AO in the OCP group was statistically significant (*p* < 0.01). The pooled-RR of AO in females not taking OCPs was not significantly different from males (*p*=0.45).

**Conclusions:**

OCP use significantly elevated the risk of AO in females. Females who did not take OCPs had a similar risk of developing AO compared to males, suggesting that OCP use is a potential effect modifier. Neither postoperative antibiotics use nor the type of postoperative analgesic significantly affected AO incidence in those taking OCPs.

## 1. Introduction

Alveolar osteitis (AO) is one of the most common postoperative complications of third molar extractions [1]. AO, referred to colloquially as dry socket, is an inflammation of the alveolar process that takes place after the alveolar bone has been exposed to the oral cavity after an extraction [2]. Typically, after a tooth extraction a thrombus forms in the empty socket, leading to uneventful healing. However, in the case of AO the blood clot is lost [2].

While the definition of AO may differ, most would agree that AO involves postoperative pain at the site of dislodgement of the blood clot. The patient often experiences severe pain, halitosis, foul taste, lymphadenopathy, and increased healing time [3]. These symptoms can last from ten to forty days [4]. AO is typically treated with saline irrigation, the application of topical anesthetics, and with regularly changed surgical dressings [3]. The incidence of AO has been reported to be 2% following routine extractions and between 20 and 30% after third molar extractions [3, 5]. Although the pathophysiology of AO is not fully understood, it is thought to be the result of fibrinolysis of the blood clot due to the action of plasmin and kinins and/or a subclinical bacterial infection present in the area at the time of surgery [4]. Notable proposed risk factors include smoking, the female sex, intake of oral contraceptives (OCPs), age, vasoconstrictors, and the menstrual cycle [2].

OCPs are thought to increase fibrinolysis by increasing serum plasminogen while decreasing serum plasminogen activator inhibitor-1 [6]. Hence, it has been stipulated that the characteristic loss of thrombus in AO may be associated with the adverse effects of OCPs on coagulation and fibrinolysis. It has been hypothesized that this may be bacteria-mediated fibrinolysis of the thrombus, hence antimicrobials such as chlorhexidine may serve as a preventative treatment for AO [7].

The primary objective of this systematic review and meta-analysis was to assess the risk of AO in OCP users. The secondary objectives included the analysis of several variables to assess their effect on the incidence of AO in OCP users, including sex, intake of preoperative and postoperative antibiotics, postoperative opioid and nonopioid analgesics, the menstrual cycle, smoking, and the decade of publication of the study. The latter in order to determine if the formulation was a determining factor.

## 2. Materials & Methods

### 2.1. Protocols and Guidelines

This review was planned and conducted in accordance with the Meta-Analysis of Observational Studies in Epidemiology (MOOSE) [8], and the Preferred Reporting Items for Systematic Reviews and Meta-Analyses (PRISMA) [9] guidelines. Investigators used the PICOT (Patient/Population, Intervention/Indicator, Compare/Controls, Outcome, Time/Type of Study or Question) search strategy tool to conduct the literature search. This protocol was not registered.

### 2.2. Eligibility Criteria

To be considered for this review, all studies were required to meet the following criteria:

- Study participants (patient/population): females who took OCPs before, after, or at the time of extraction compared to females who did not take OCPs, all of whom required (intervention) extraction of their third molar(s). No age, race, or health restrictions were applied.
- Type of Study: Observational Human Studies (Case-Controls, Cross-Sectional, Prospective and Retrospective Cohorts) and Clinical Trials were selected for the purpose of this study. Case Reports, Case Series, Letters to the Editor, Personal Communications, and Qualitative Studies were excluded.
- Outcome: AO was clinically diagnosed by postoperative pain and total or partial loss of the blood clot at the site of extraction 1–7 days following surgery.
- Reporting of results: studies were only eligible if they reported the frequency and a measure of association (Odds Ratio (OR), and/or Relative Risk (RR) with a 95% confidence interval (CI)) with AO cases among OCP users and nonusers (Controls).
- Length of OCP use (Time): no restrictions applied.
- Accessibility of data: studies were only eligible if they were published as full publications in the English language.

### 2.3. Literature Search

The literature search was conducted using PubMed/MEDLINE, EMBASE, and Cochrane Library databases, via the following search terms: (combined oral contraceptives [MeSH Terms]) OR (oral contraceptive agents, hormonal [MeSH Terms]) AND (alveolar osteitis [MeSH Terms]) OR (dry socket [MeSH Terms]) until March 2022. All search terms were adapted for each database, as necessary. To ensure completeness, the references in the articles selected for inclusion were searched manually and independently by investigators (MT, DGA, and JAS) for potentially relevant articles.

### 2.4. Study Selection & Investigators

Two investigators (MT and DGA) performed independent article searches that met the eligibility criteria. Publications were further examined based on the full title and published abstract. At this point, duplicates and articles that did not meet the eligibility criteria were removed, and a full-text article was obtained for those deemed eligible for inclusion. Once completed, the investigators compared the results, and any discrepancies were resolved by a third investigator (JAS).

### 2.5. Data Collection

Two investigators (MT and DGA) independently extracted data using a data abstraction form including studies' characteristics such as the country in which the study was conducted, population characteristics (mean age, sex), study design, length of follow-up, exposure (OCP use), time to outcome measure (development of AO), and strength of association. Any discrepancies in the data abstraction were reviewed and resolved by a third investigator (JAS).

### 2.6. Data Analysis

The meta-analysis was conducted using the “meta” package in R version 4.0.2 [10]. The data were analyzed using the Mantel-Haenszel method. Incidence rates among OCP users and non-OCP users were used to calculate pooled-RR using the random or fixed effects model based on the heterogeneity among the selected studies.

### 2.7. Assessment of Heterogeneity

The degree of dissimilarity in the results of individual studies (or heterogeneity) was assessed graphically using forest plots, and the exact binomial CIs were calculated. Statistical heterogeneity between the reviewed studies was quantified with the DerSimonian and Laird estimate (I^2^), where I^2^ > 30%, I^2^ > 50%, and I^2^ > 75% indicated moderate, substantial, and considerable heterogeneity, respectively. Cochran's *Q* test was used in conjunction with a forest plot to determine the significance (*p* ≤ 0.05) of the calculated heterogeneity between studies.

#### 2.7.1. Quality Assessment

The quality of the selected studies was assessed independently by three investigators (MT, DGA, JAS) with the “Quality Assessment Tool for Observational Cohort and Cross-Sectional Studies” of the National Institute of Health (NIH) [11]. All studies were assessed for potential risk of selection bias. Based on the results of this assessment, studies were rated as good (“+”) or poor (“−”), indicating a high or low risk of bias, respectively (Table 1).

#### 2.7.2. Risk of Bias across the Studies

The risk of publication bias was assessed by visual analysis of a funnel plot [28], where a visually symmetrical plot indicated a low risk of publication bias and a visually asymmetrical plot indicated a high risk of publication bias.

### 2.8. Subgroup and Sensitivity Analyses

Subgroup analyses were conducted based on intake of postoperative antibiotics and postoperative opioid or nonopioid analgesics, smoking status, the decade of publication of the study, and menstrual cycle.

## 3. Results

### 3.1. Study Selection

**Figure 1:**
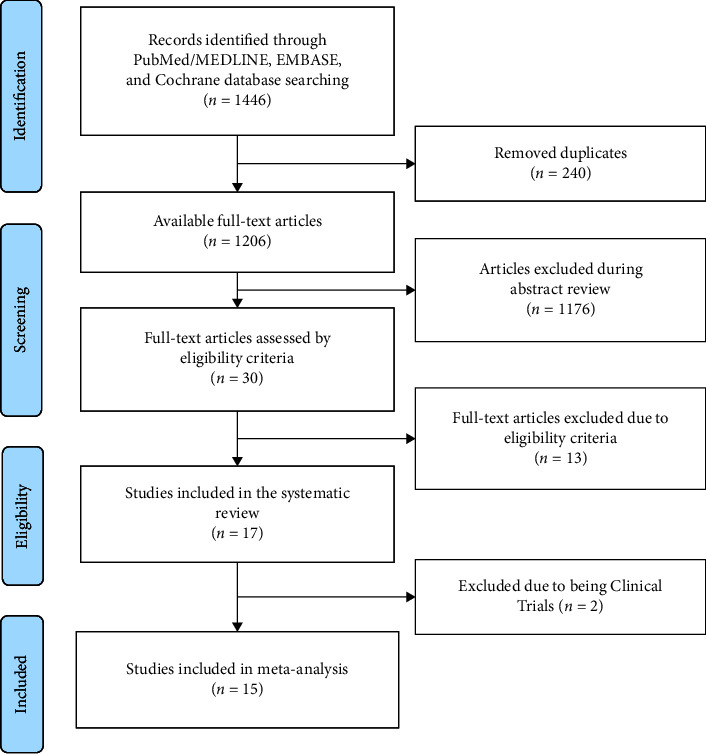
PRISMA flow chart.

PubMed/MEDLINE, EMBASE, and Cochrane Libraries identified 1446 articles that met the inclusion criteria, 240 of which were duplicates. 1176 articles were further eliminated after abstract review. The remaining thirty were reviewed by full-text, of which seventeen were deemed eligible for the systematic review and fifteen for the meta-analysis (Figure 1).

### 3.2. Study Characteristics

The included seventeen [7, 12–27] studies were published between 1974 and 2019. Thirteen [12–22, 26, 27] were prospective studies; one [23] was cross-sectional, one [25]was retrospective, and two [7, 24] were clinical trials. The systematic review included all seventeen studies [7, 12–27], and for the purpose of the meta-analysis, the two clinical trials were eliminated from the analysis, yielding a total of fifteen studies. It is worth noting that twelve [7, 12, 13, 16–18, 20, 21, 23, 25–27] studies included male participants as well (Table 2).

### 3.3. Quality and Risk of Bias Assessment

**Figure 2:**
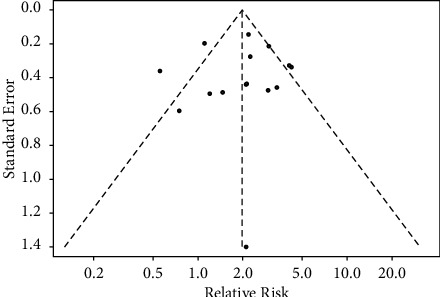
Funnel plot of studies included in the meta-analysis.

All included studies were of good methodological quality (Table 1), and the funnel plot of the included studies was visually symmetrical (Figure 2), indicating a low risk of publication bias.

### 3.4. AO in Females (OCP Users vs. Non-OCP Users)

**Figure 3:**
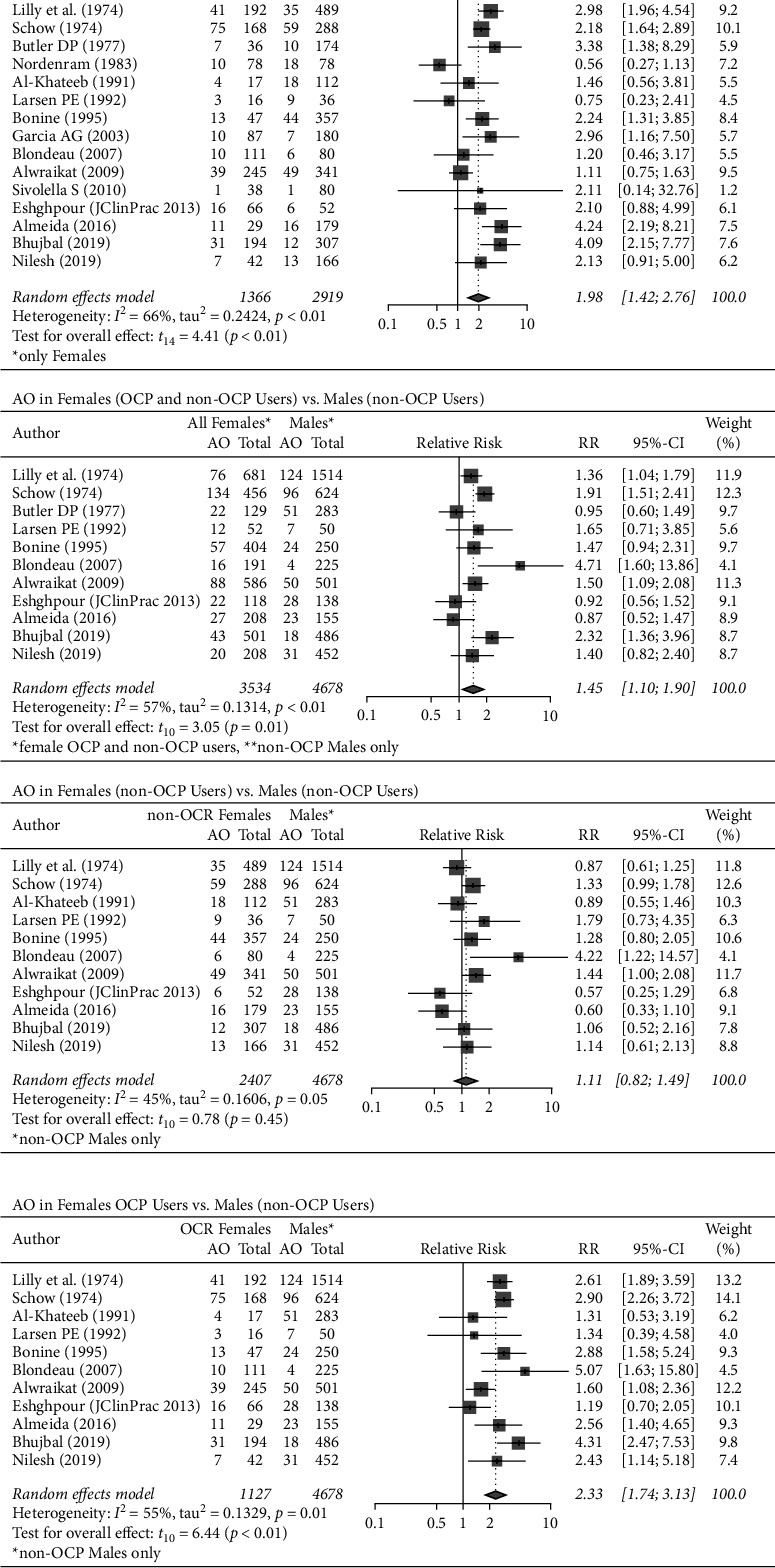
AO among OCP users analysed by sex.

In the fifteen [12–23, 25–27] included studies, 581 out of 4285 females presented with AO. Of these, 1366 (31.88%) females took OCPs, and 2919 (68.12%) did not. Females who took OCPs had approximately two times (pooled-RR: 1.98, 95% CI: 1.42–2.76) the risk of developing AO when compared to female non-OCP users, and this difference was statistically significant (*p* < 0.01). There was substantial heterogeneity (I^2^ = 66%, *p* < 0.01) across the studies (Figure 3).

#### 3.4.1. AO in Females (OCP and Non-OCP Users) vs. Males (Non-OCP Users)

Eleven [12, 13, 16–18, 20, 21, 23, 25–27] studies included both male and female participants (3534 females and 4678 males). The pooled estimate showed that females were 1.5 times (pooled-RR: 1.45, 95% CI: 1.10–1.90) more likely to develop AO when compared to males, and this difference was statistically significant (*p*=0.01). There was substantial heterogeneity across the twelve studies (I^2^ = 57%, *p* < 0.01) (Figure 3).

#### 3.4.2. AO in Female Non-OCP Users vs. Males (Non-OCP Users)

Eleven [12, 13, 16–18, 20, 21, 23, 25–27] studies were included in this analysis, and the pooled estimate showed that female non-OCP users (*n* = 2407) were 1.1 times (pooled-RR: 1.11, 95% CI: 0.82–1.49) more likely to develop AO following a third molar extraction when compared to male non-OCP users (*n* = 4678). This difference was not statistically significant (*p*=0.45). There was moderate heterogeneity across the twelve studies (I^2^ = 45%, *p*=0.05) (Figure 3).

#### 3.4.3. AO in Female OCP Users vs. Males (Non-OCP Users)

Similarly, when comparing female OCP users (*n* = 1127) with males (*n* = 4678), the analysis showed that OCP users were more than two times as likely (pooled-RR: 2.33, 95% CI: 1.74–3.13) to develop AO following third molar extraction. This difference was statistically significant (*p* < 0.01). There was substantial heterogeneity across the twelve studies (I^2^ = 55%, *p* < 0.01) (Figure 3).

### 3.5. Subgroup Analysis of AO among Female OCP Users Who Were Prescribed Postoperative Analgesics

**Figure 4:**
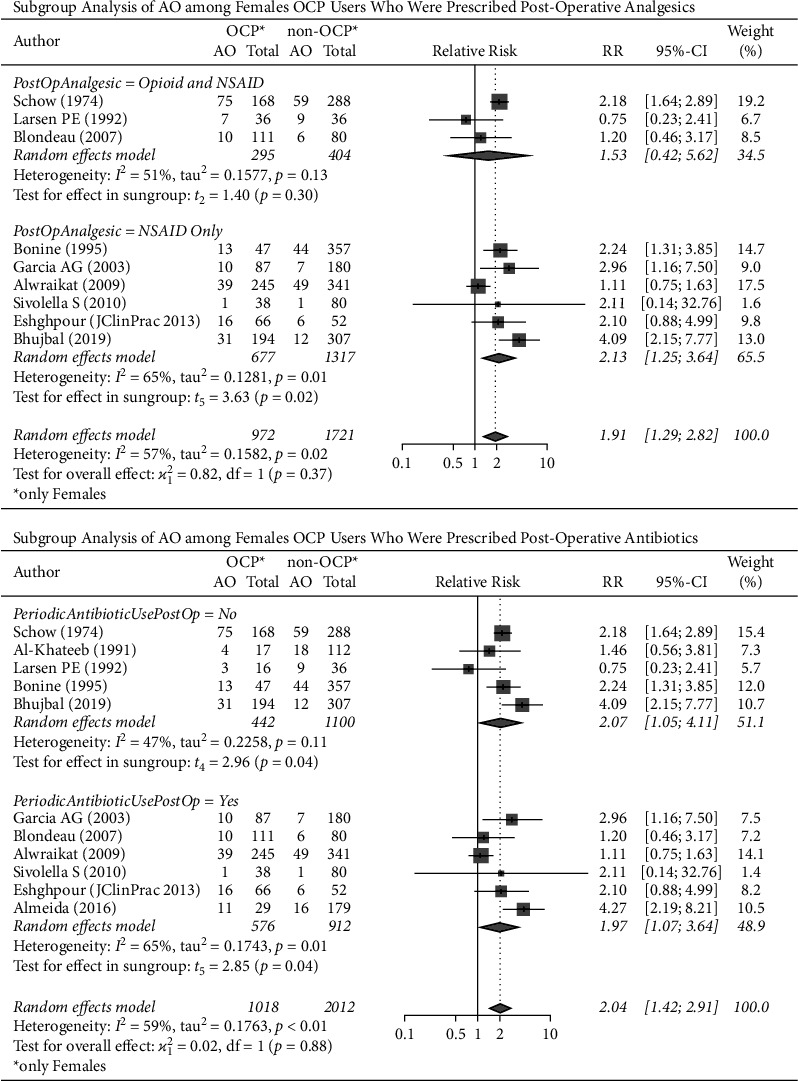
AO and OCPs by analgesics and antibiotics use.

The female participants in six [18, 19, 21, 22, 24, 26] of the included studies (677 OCP users and 1317 non-OCP users) were prescribed postoperative nonopioid analgesics after third molar extraction, while only three [13, 17, 20] studies included participants (295 OCP users and 404 non-OCP users) who were prescribed both nonopioid and opioid analgesics. Females taking OCPs and nonopioid analgesics had more than twice (pooled-RR: 2.13, 95% CI: 1.25–3.64, *p*=0.02) the chance of developing AO when compared to females who did not take OCPs, while females who took OCPs, nonopioid analgesics, and opioids had a pooled-RR of 1.53 (pooled-RR: 1.53, 95% CI: 0.42–5.62, *p*=0.30). The difference in the pooled estimate between the two subgroups was not statistically significant (*p*=0.37). There was substantial heterogeneity among studies (I^2^ = 57%, *p*=0.02) (Figure 4).

### 3.6. Subgroup Analysis of AO among Female OCP Users Who Were Prescribed Postoperative Antibiotics

The female participants of six [19–22, 25] of the studies (576 OCP users and 912 non-OCP users) were given postoperative antibiotics and were compared with the participants of five [13, 16–18, 26] of the studies who were not (442 OCP users and 1100 non-OCP users).

The analysis showed that females who took OCPs had more than twice (pooled-RR: 2.07, 95% CI: 1.05–4.11, *p*=0.04) and approximately twice (pooled-RR: 1.97, 95% CI: 1.07–3.64, *p*=0.04) the risk of developing AO following a third molar extraction when compared to females who did not take OCPs in regard to postoperative antibiotic use “no” and “yes,” respectively. The difference between the two subgroups (“no” vs. “yes”) was not statistically significant (*p*=0.88), and there was substantial heterogeneity among studies (I^2^ = 59%, *p* > 0.01) (Figure 4).

## 4. Discussion

### 4.1. Risk of AO in Females (OCP Users vs. Non-OCP Users)

This meta-analysis included fifteen studies and found that OCP use was a significant risk factor for AO. The two clinical trials that were excluded from the meta-analysis also found that female OCP users were more likely to develop AO. In this study, females taking OCPs were approximately twice as likely to develop AO following third molar extractions compared to females not taking OCPs (Figure 3).

### 4.2. Incidence of AO by Sex

The female sex is an often-mentioned risk factor for AO. However, the correlation is controversial, and OCP use remains an important effect modifier [2]. The evidence is controversial, and while the majority of the studies have found no significant differences between the sexes [24, 25, 29], Blondeau and Daniel [20] found a significantly higher incidence among women. This study found that the incidence of AO was highest among those taking OCPs, with 1 in 5 (20.95%) females taking OCPs developing AO following a third molar extraction. Females overall had a higher pooled incidence of AO than males. 1 in 7 (13.73%) females developed AO, while 1 in 10 (10.78%) males developed AO following third molar extraction. These findings suggest that OCP use is a significant effect modifier that explains the increased pooled incidence of AO in females overall. When controlled for OCP use, the female sex was not a risk factor for AO.

The pooled risk for females when controlled for OCP use was not significantly different from the males (pooled-RR = 1.11, *p*=0.41), further suggesting that OCP is a risk factor for AO.

A recent meta-analysis by Bienek and Filliben [30] examined the occurrence of AO in OCP users. The authors showed a higher pooled-RR in females taking OCPs when compared to males, and an increased pooled-RR in females taking OCPs when compared to those not taking them. These findings are in support of this study, and both found a pooled-RR of 1.1–1.2 for AO in females not taking OCPs versus males, where Bienek and Filliben [30] found this difference to be statistically significant, and this study did not (*p*=0.45). Bienek and Filliben [30] included studies providing data on AO after the extraction of any tooth, whereas this study included only AO after the third molar extraction, which may explain the difference in significance in the analysis [30]. Further investigation into the effect of sex on AO independent of OCP use is needed.

### 4.3. Time Period

Rosendaal et al. [31] noted that the formulations of OCPs have changed over time due to emerging evidence linking OCPs to venous thrombosis; this side effect has been attributed to the use of estrogen in their formulation. Therefore, the concentration of estrogen has been decreased over the years to reduce the likelihood of complications [32], and some have speculated that this decrease may have an effect on the incidence of AO [20, 33]. A subgroup analysis was conducted according to the decade of publication to assess this effect. There was no significant trend between the time period and the pooled-RR of the incidence of AO in OCP users (Supplement 1).

### 4.4. Postoperative Analgesics in Female OCP Users

The pooled-RR of AO was 2.13 times higher in females who were taking OCPs compared to non-OCP users when prescribed postoperative NSAIDs only. Female participants who took both postoperative opioids and NSAIDs had a pooled-RR of 1.53 times higher than non-OCP users (Figure 4).

### 4.5. Postoperative Antibiotics in Females Taking OCPs

There is no clear consensus on the use of prophylactic antibiotics for the prevention of AO [34], and there are concerns over potential side effects and microbial resistance associated with antibiotic use [35]. Notably, the postoperative use of a chlorhexidine rinse in preventing AO is well-supported in the literature [7, 36, 37].

Only one of the studies [19] included in this meta-analysis collected data on the incidence of AO in patients that took antibiotics preoperatively. The authors found no significant differences in the incidence of AO between those who took antibiotics preoperatively and those who did not.

On the other hand, the pooled-RR of AO was approximately two times higher in those taking OCPs and postoperative antibiotics than in females only taking postoperative antibiotics and in those taking OCPs without postoperative antibiotics than in females taking only postoperative antibiotics (Figure 4). The difference between the two subgroups was not statistically significant (*p*=0.88), suggesting that postoperative antibiotics do not offset the increased incidence of AO associated with OCP use.

### 4.6. Menstrual Cycle

Hormonal changes, particularly in estrogen, precipitated by the menstrual cycle may have an effect on the incidence of AO. Nordenram and Grave [15] suggested that changes in plasminogen activators in the saliva throughout the menstrual cycle may affect the incidence of AO. Two of the included studies investigated the relationship between the menstrual cycle phase and the incidence of AO [15, 23]. The findings of Eshghpour and Nejat [23] suggest an increased risk of AO associated with third molar extractions on days 8–21 of the menstrual cycle that is increased with OCP use. In contrast, Nordenram and Grave [15] suggest that the incidence of AO is lowest on day 14 of the menstrual cycle and suggest performing the extractions when the female patient is not menstruating and should be deferred until an OCP withdrawal period in users. Current evidence pertaining to the effect of the menstrual cycle phase on the incidence of AO is inconclusive (Supplements 2–4), and further research is needed to determine if an association exists.

### 4.7. Insufficient Evidence for AO, OCP Use, and Smoking

The included studies did not provide sufficient evidence on how OCP use and concurrent smoking are associated with AO. Studies were subgrouped by whether smokers were included in the study or not (Supplement 5). The included studies did not provide data on the number of concurrent smokers and OCP users that participated, so the ratio of concurrent users, smokers only, OCP use only, and nonusers was unknown. Further research is needed to determine whether smoking is associated with a change in risk for AO in women taking OCPs.

## 5. Conclusion

This meta-analysis was able to confirm that the intake of oral contraceptives (OCP) is a risk factor for alveolar osteitis (AO) after third molar extraction. According to the CDC, in 2015–2017, 64.9% of the 72.2 million women aged 15–49 in the US were currently using contraception, 12.6% of which were using oral contraceptive pills. We believe that the results of our study are impactful at a predoctoral and postdoctoral level and useful for interdisciplinary and continuing education purposes, as this is something both general dentists and specialists should be aware of. The high prevalence of women using OCPs makes this a relevant finding for the care of our patients.

A dose-response relationship may exist between the hormone(s) concentration in OCPs and the incidence of AO, and the menstrual cycle phase the patient is in during the extraction may be a risk factor. This paper will provide other researchers with the premise for potential further research regarding the topic.

The difference between females not taking OCPs and males was not significant, suggesting that the female sex is not a risk factor for AO. OCP use was likely an effect modifier in previous studies. Those considering third molar extraction should be advised to temporarily discontinue OCPs prior to surgery. Further investigation should be conducted to evaluate how far prior to surgery OCPs should be discontinued to minimize the risk of AO. Despite the decreasing quantity of estrogen in OCP formulations over time, this meta-analysis did not find evidence that supports the theory that the decreasing quantity of estrogen in OCP formulations would result in a decreased incidence of AO. Neither the type of analgesic taken nor taking antibiotics postoperatively had a significant effect on the incidence of AO. The evidence supporting the menstrual cycle phase as a risk factor for AO was limited.

## Figures and Tables

**Table 1:** Quality assessment tool for selected studies in this systematic review.

Q	Lilly 1974 [12]	Schow 1974 [13]	Butler 1977 [14]	Nordenram 1983 [15]	Al-Khateeb 1991 [16]	Larsen 1992 [17]	Bonine 1995 [18]	Hermesch 1998 [7]	Garcia 2003 [19]	Blondeau 2007 [20]	Alwraikat 2009 [21]	Sivolella 2010 [22]	Eshghpour 2013 [23]	Eshghpour 2013 [24]	Almeida 2016 [25]	Bhujbal 2019 [26]	Nilesh 2019 [27]
1	+	+	+	+	+	+	+	+	+	+	+	+	+	+	+	+	+
2	+	+	+	+	+	+	+	+	+	+	+	+	+	+	+	+	+
3	+	+	+	+	+	+	+	+	+	+	+	+	+	+	+	+	+
4	+	+	+	+	+	+	+	+	+	+	+	+	+	+	+	+	+
5	−	+	+	+	−	+	+	+	+	+	+	+	+	−	+	−	−
6	+	−	+	+	+	+	+	+	+	+	+	+	+	+	+	+	+
7	+	+	+	+	+	+	+	+	+	+	+	+	+	+	+	+	+
8	+	+	+	+	+	+	+	+	+	+	+	+	+	+	+	+	+
9	+	+	+	+	+	+	+	+	+	+	+	+	+	+	+	+	+
10	−	−	+	−	+	−	+	+	+	+	−	−	+	+	−	−	−
11	+	+	+	+	+	+	+	+	+	+	+	+	+	+	+	+	+
12	+	−	−	−	−	+	−	+	−	−	−	−	−	+	−	+	+
13	+	+	+	+	+	+	+	+	+	+	+	+	+	+	+	+	+
14	+	−	−	−	+	−	−	+	+	−	−	−	−	+	−	+	+

Q: Question, “+” = low risk/good quality; “−” high risk/poor quality. 1, was the research question or objective in this paper clearly stated? 2, was the study population clearly specific and defined? 3, was the participation rate of eligible persons at least 50%? 4, were all the subjects selected or recruited from the same or similar populations (including the same time period)? 5, was a sample size justification, power description, or variance and effect estimates provided? 6, for the analyses in this paper, were the exposure(s) of interest measured prior to the outcome(s) being measured? 7, was the timeframe sufficient so that one could reasonably expect to see an association between exposure and outcome if it existed? 8, for exposures that can vary in amount or level, did the study examine different levels of the exposure as related to the outcome (e.g., categories of exposure or exposure)? 9, were the exposure measures (independent variables) clearly defined, valid, reliable, and implemented consistently across all study participants? 10, was the exposure(s) assessed more than once over time? 11, were the outcome measures (dependent variables) clearly defined, valid, reliable, and implemented consistently across all study participants? 12, were the outcome assessors blinded to the exposure status of participants? 13, was the loss to follow-up after baseline 20% or less? 14, were key potential confounding variables measured and adjusted statistically for their impact on the relationship between exposure(s) and outcome(s)?

**Table 2:** Characteristics of the selected studies in this systematic review.

Authors	Year	Study design	Study design total incidence of AO					
			OCP users, females		Non-OCP user, male and females		Non-OCP users, females	
			*n*	(%)	*n*	(%)	*n*	(%)
Lilly et al., [12]	1974	Prospective	192	21.4	975	7.9	489	7.2
Schow et al., [13]	1974	Prospective	168	44.6	912	17.0	288	20.5
Butler et al., [14]	1977	Prospective	36	19.4	—	—	174	5.7
Nordenram et al., [15]	1983	Prospective	78	12.8	—	—	78	23.1
Al-Khateeb et al., [16]	1991	Prospective	17	23.5	395	17.5	112	16.1
Larsen et al., [17]	1992	Prospective	16	18.8	86	18.6	36	25.0
Bonine et al., [18]	1995	Prospective	47	27.7	607	11.2	357	12.3
Hermesch et al., [7]	1998	Clinical trial	59	35.6	212	20.8	111	21.6
Garcia et al., [19]	2003	Prospective	87	11.5	—	—	180	3.9
Blondeau et al., [20]	2007	Prospective	111	9.0	305	3.3	80	7.5
Alwraikat et al., [21]	2009	Prospective	245	15.9	842	11.8	341	14.4
Sivolella et al., [22]	2010	Prospective	38	2.6	—	—	80	1.3
Eshghpour et al., [23]	2013	Cross-sectional	66	24.2	190	17.9	52	11.5
Eshghpour et al., [24]	2013	Clinical trial	132	29.5	—	—	158	22.5
Almeida et al., [25]	2016	Retrospective	29	37.9	334	11.7	179	8.9
Bhujbal et al., [26]	2019	Prospective	194	16.0	793	3.8	307	3.9
Nilesh et al., [27]	2019	Prospective	42	16.3	618	7.1	166	6.9

## Data Availability

The data supporting this systematic review and meta-analysis are from previously published studies (all have been cited in this manuscript). The processed data are available in the tables and figures and supplement files that are being submitted along with the manuscript.

## Supplementary Materials

Supplementary Materials Supplement 1: subgroup analysis according to decade of publication. Supplement 2: subgroup analysis of AO in females (OCP users and non-OCP users) at days 1 and 14 of their menstrual cycle. Supplement 3: subgroup analysis of AO in female non-OCP users by menstrual cycle. Supplement 4: subgroup analysis of AO in females taking OCPs by menstrual cycle. Supplement 5: subgroup analysis of AO and OCP by smoking.
