# Supplementary figures and images for: Oral Contraceptive Use and Alveolar Osteitis Following Third Molar Extraction: A Systematic Review and Meta-Analysis
Source: Int J Dent. 2022 Nov 1;2022:7357845. doi: 10.1155/2022/7357845 (PMC9643054; doi:10.1155/2022/7357845)

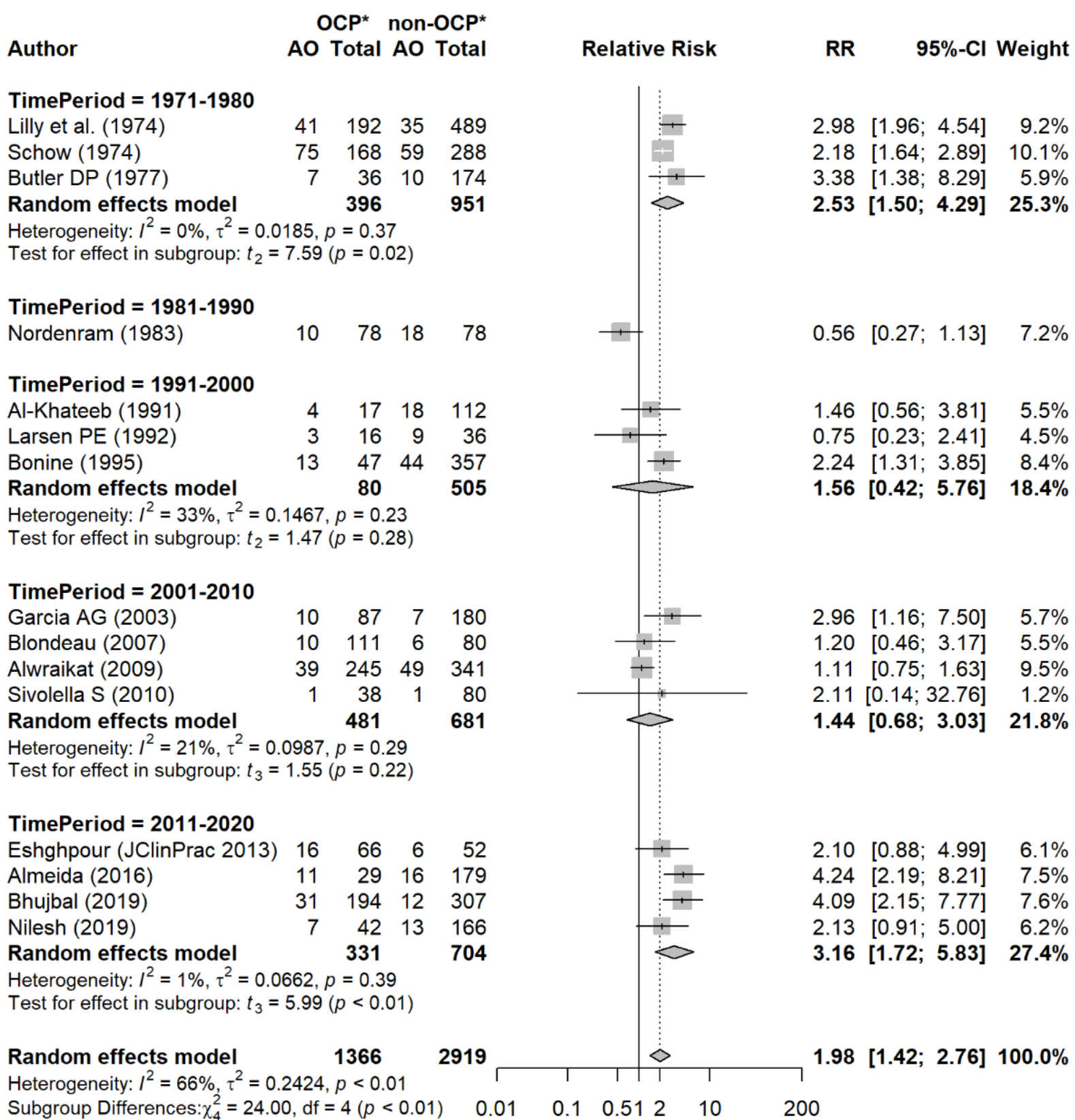

\*only Females

Supplement: Supplementary Materials — Supplement 1: subgroup analysis according to decade of publication. Supplement 2: subgroup analysis of AO in females (OCP users and non-OCP users) at days 1 and 14 of their menstrual cycle. Supplement 3: subgroup analysis of AO in female non-OCP users by menstrual cycle. Supplement 4: subgroup analysis of AO in females taking OCPs by menstrual cycle. Supplement 5: subgroup analysis of AO and OCP by smoking. [file 7357845.f1.zip › Supplement 1 New Time period subgroup IJD e.pdf]

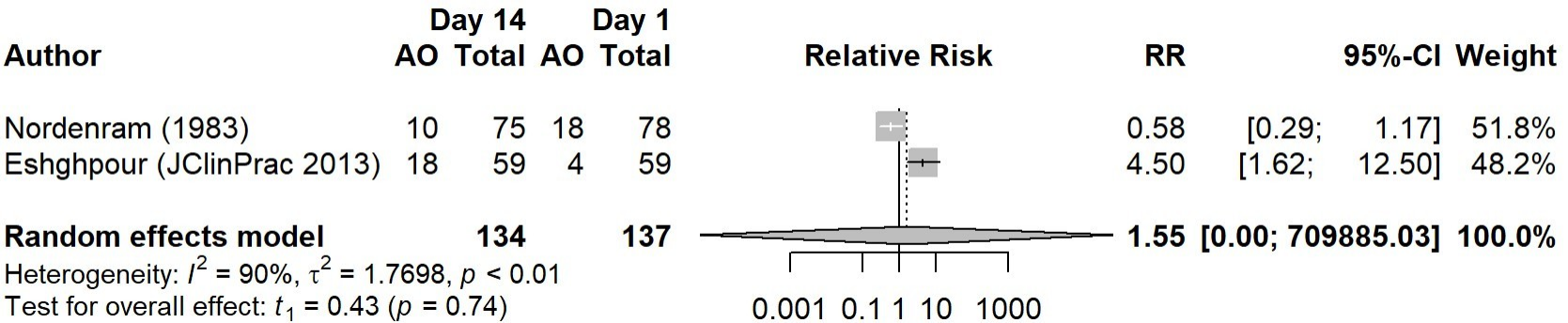

Supplement: Supplementary Materials — Supplement 1: subgroup analysis according to decade of publication. Supplement 2: subgroup analysis of AO in females (OCP users and non-OCP users) at days 1 and 14 of their menstrual cycle. Supplement 3: subgroup analysis of AO in female non-OCP users by menstrual cycle. Supplement 4: subgroup analysis of AO in females taking OCPs by menstrual cycle. Supplement 5: subgroup analysis of AO and OCP by smoking. [file 7357845.f1.zip › Supplement 2 New Female Total Menstrual IJD e.pdf]

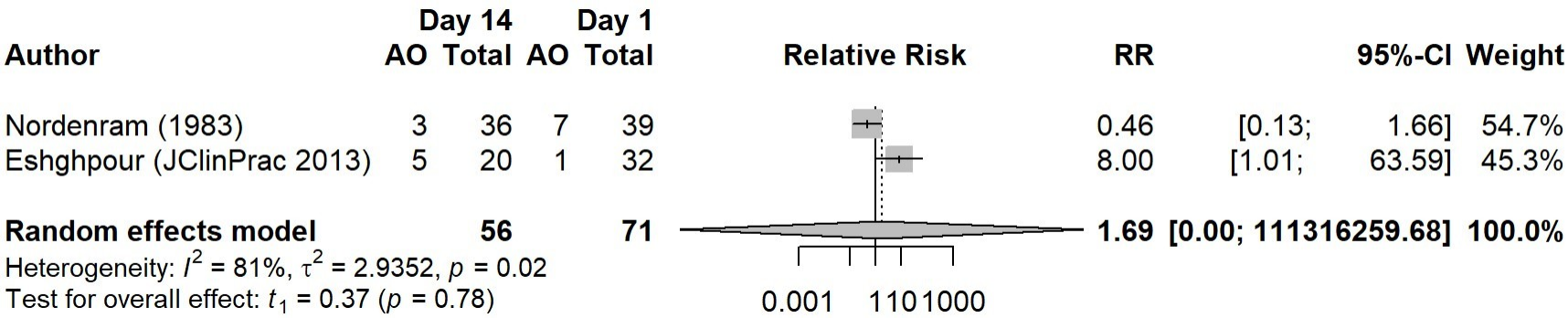

Supplement: Supplementary Materials — Supplement 1: subgroup analysis according to decade of publication. Supplement 2: subgroup analysis of AO in females (OCP users and non-OCP users) at days 1 and 14 of their menstrual cycle. Supplement 3: subgroup analysis of AO in female non-OCP users by menstrual cycle. Supplement 4: subgroup analysis of AO in females taking OCPs by menstrual cycle. Supplement 5: subgroup analysis of AO and OCP by smoking. [file 7357845.f1.zip › Supplement 3 New No OCP Menstrual Subgroup IJD e.pdf]

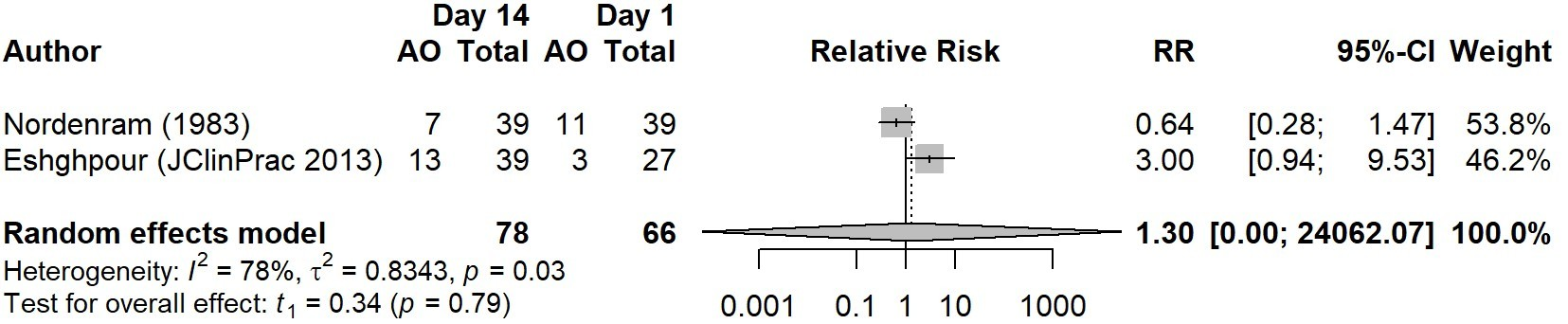

Supplement: Supplementary Materials — Supplement 1: subgroup analysis according to decade of publication. Supplement 2: subgroup analysis of AO in females (OCP users and non-OCP users) at days 1 and 14 of their menstrual cycle. Supplement 3: subgroup analysis of AO in female non-OCP users by menstrual cycle. Supplement 4: subgroup analysis of AO in females taking OCPs by menstrual cycle. Supplement 5: subgroup analysis of AO and OCP by smoking. [file 7357845.f1.zip › Supplement 4 New OCPs Menstrual Subgroup IJD e.pdf]

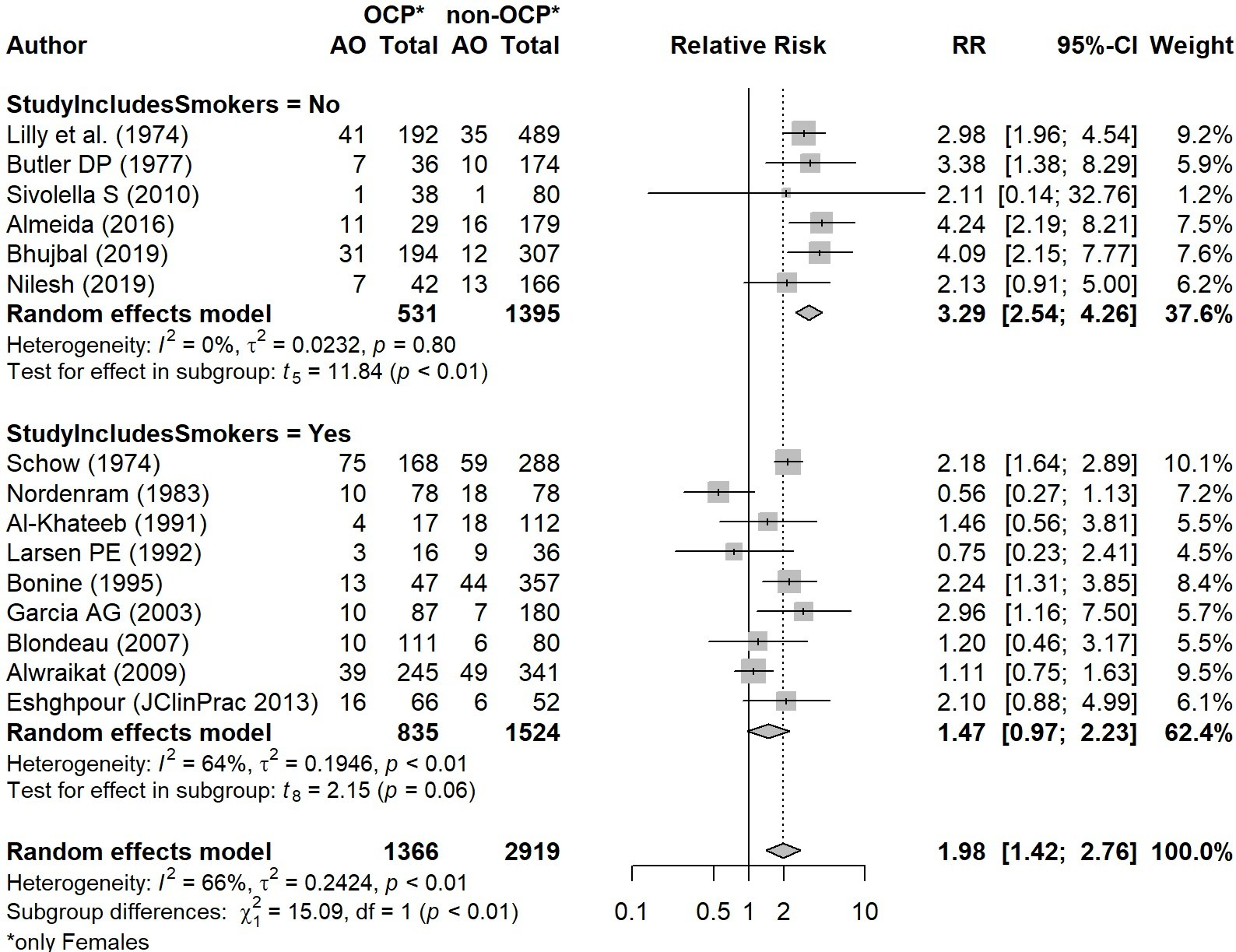

Supplement: Supplementary Materials — Supplement 1: subgroup analysis according to decade of publication. Supplement 2: subgroup analysis of AO in females (OCP users and non-OCP users) at days 1 and 14 of their menstrual cycle. Supplement 3: subgroup analysis of AO in female non-OCP users by menstrual cycle. Supplement 4: subgroup analysis of AO in females taking OCPs by menstrual cycle. Supplement 5: subgroup analysis of AO and OCP by smoking. [file 7357845.f1.zip › Supplement 5 New Smoking Subgroup IJD e.pdf]
